# An evaluation of the relationship between clinical requirements and tests of competence in a competency-based curriculum in dentistry

**DOI:** 10.1186/s12909-023-04438-3

**Published:** 2023-08-18

**Authors:** Shivaughn M. Marchan, Larry G. Coldero, William A.J. Smith

**Affiliations:** 1 Unit of Restorative Dentistry, School of Dentistry, The University of the West Indies, St. Augustine, Trinidad and Tobago; 2 Unit of Restorative Dentistry, School of Dentistry, The University of the West Indies, St. Augustine, Trinidad and Tobago; 3 School of Dentistry, The University of the West Indies, St. Augustine, Trinidad and Tobago

**Keywords:** Dentistry, Minimal clinical requirements, Competence

## Abstract

**Background:**

The development of competencies in dentistry is a complicated process that calls for the development of not just cognitive and psychomotor abilities but also behaviors and attitudes that change as technical proficiency increases and meaningful patient encounters occur. This study examined the relationship between the number of clinical requirements completed by dental students and subsequent performance on tests of competence. The null hypothesis stated there would be no significant linear relationship different from zero between absolute clinical requirements and grades attained in various tests of clinical competence.

**Methods:**

Retrospective assessment data for 81 students were used in this analysis. Data included the amounts of clinical requirements completed for operative dentistry, endodontics, periodontics, and fixed prosthodontics together with data on the respective performance in tests of competence. Correlation was ascertained between grades for tests of competence and the corresponding clinical requirements using a non-parametric Spearman’s Rho test at an alpha level of 0.05.

**Results:**

Fixed prosthodontics and posterior endodontics were the least common procedures completed by dental students. Statistically significant weak correlations were found between the amounts of clinical requirements performed for posterior endodontic(p = 0.005) and operative procedures (p = 0.006) and associated performance in tests of competence. A moderate correlation was found between the number of fixed prosthodontic procedures completed and associated performance in tests of competence. This latter correlation, however, was not statistically significant (p = 0.654). A significant weak correlation was found between requirements completed for periodontics and the associated test of competence (p = 0.04). A highly statistically significant moderate correlation was found between clinical requirements for anterior endodontics and the associated performance in the tests of competence (p < 0.001).

**Conclusion:**

The null hypothesis was rejected since a positive correlation was found between the absolute clinical requirements completed and grades in tests of competence. However, only a weak to moderate degree of correlation was found between the completion of clinical requirements and performance in tests of competence for common clinical procedures that new dental graduates should be able to perform.

## Background

Competency-based curricula have been widely accepted as a framework in dental education [1]. There are four main tenets of competency-based education: a focus on required learning outcomes, an emphasis on the acquisition and development of abilities or skills, a concomitant reduction of time-based or quota-based training and the promotion of student-centered learning strategies [2]. The development of competencies in dentistry is a complex process that requires not only the development of cognitive and psychomotor skills but the development of behaviors and attitudes that evolve because of increasing technical competence as meaningful interactions with patients take place.

Synonymous with competency-based education in dentistry has been the shift that individual dental students must be responsible for the holistic and comprehensive management of patients under their care. Training programs must respond to such shifts by identifying what is crucial for safe and competent practice and design learning activities and experiences so that students may graduate as safe and qualified beginners [3]. This development of competence starts with requisite pre-clinical courses before the management of patients. This shift also requires dental schools to provide students with appropriate patients with complex dental needs, where continued interaction with patients will allow novice clinical dental students to develop the required competencies [3].

Central to competency-based education, many dental schools have moved away from quota-based training and instead have described the minimum experience thresholds that students must fulfil, so-called minimal clinical requirements, in the development of competencies [4]. Conversely, some schools have no such requirements. A recent scoping review revealed approximately 60% of dental schools in the United Kingdom did not mandate minimum clinical requirements and described the possibility of students graduating without certain clinical experiences [4].

A school of dentistry in the English-speaking Caribbean adopted a competency-based curriculum in 2000 [5]. Students must meet minimum clinical experiences and be successful in competency assessments to progress from year to year or to be eligible for the final exit examination and graduation. Some students however exceed these minimum clinical experiences. At this school, an absolute number of two procedures per clinical discipline have been adopted as the minimum clinical experience or clinical requirements. This adopted minimum clinical experience aligns with the perception of reduced clinical requirements of competency-based education held by dental faculty in North American dental schools [6].

The use of minimal clinical requirements takes a work-oriented approach to the development of competence. The attributes that dental learners have regarding the clinical management of patients serve as the starting point for proponents of a work-oriented approach in the development of competence. Work-oriented techniques start with the tasks that need to be completed that are then translated into learning activities and the subsequent development of attributes required to complete said learning activities [7].

The purpose of this research, which used retrospective student assessment data explored the relationship between absolute clinical experiences and performance in tests of competence. The null hypothesis stated there would be no significant linear relationship different from zero between absolute clinical requirements and grades attained in various tests of clinical competence.

## Methods

Ethical approval for the study was granted by the university’s Ethics Committee [CREC-SA.1052/06/2021]. Retrospective student assessment data for all students in the graduating classes of 2017, 2018, and 2019 were included together with data on minimal clinical requirements (clinical requirements) for students of these graduating classes. Data were de-identified and aggregated for statistical analysis. Clinical experiences for operative procedures, endodontics (single and multi-rooted teeth), fixed prosthodontics, and periodontics were included. Minimum clinical experiences were counted once a clinical procedure was deemed to be completed for those procedures requiring multiple visits.

Despite an overall competency-based curriculum individual tests of competence are required for progression from year to year. A skilled clinical operative test (SCOT I) where students must demonstrate skill in the operative management of proximal caries serves as a pathway examination from the fourth to the fifth year of training. There is an equivalent test of competence in endodontics where students must either demonstrate clinical skill and associated behaviors in the endodontic management of a single canal anterior or premolar tooth, from access to master apical cone fit (SCOT II) or access, determination of the number of canals, corrected working length, and complete instrumentation of a posterior multi-rooted tooth. This examination takes place in the first semester of the fifth year.

A clinical examination in periodontology (CAP) takes place in the first semester of the fifth year. Students must demonstrate periodontal screening, history taking, full periodontal assessment, diagnosis and management strategies. For the psychomotor skills portion of the competency examination, students must detect and remove calculus, plaque and stains from teeth via scaling and or root planning using hand instruments. A SCOT-III, where students must demonstrate psychomotor hand skills by preparing abutment teeth for a 3-unit fixed bridge and fabrication of a provisional bridge is completed in the first semester of the fifth year. Except for the SCOT-III, all assessments take place on live patients who are themselves under the care of the student. These assessments all take place during the clinical phase of training and students are not required or mandated to practice on typodonts before any of these assessments since students are expected to have completed the minimum clinical requirements before the assessment or working towards completing these requirements. The successful completion of pre-clinical courses, inclusive of exercises on typodonts, is used as the benchmark to progress to clinical training only and is not considered or counted as a pre-requisite to be eligible for the clinical test of competence.

Rubrics using clinical criteria are used by assessors to facilitate objective scoring. Additionally, a minimum of two faculty assessors allows for the robust assessment of complex competencies. The final grade given for these competency assessments is a global grade, where several individual competencies were assessed during the management of the patient.

All tests of competencies, except SCOT-III, described in the foregoing paragraphs are authentic assessments where students apply didactic and experiential knowledge and psychomotor skills to the real-life problem of patient management. Patients are first pre-approved and these assessments require students to manage clinical patients in a dental setting. Students are assessed on psychomotor skill performance, professional attitudes and behaviors and application of appropriate supporting knowledge in the management of a patient in a realistic setting without assistance. For example, the SCOT I test will include a composite assessment of the presentation of the patient inclusive of medical, social and dental history, the administration of local anaesthesia, the removal of carious tooth structure, final cavity design, selection and application of an appropriate restorative material and the finished and polished restoration.

Retrospective data for 81 students were included in this analysis. Data were entered into SPSS Version 28 (IBM, Chicago) and a one-sample Kolmogorov-Smirnov test of normalcy was used for each of the variables. This was followed by a non-parametric Spearman’s Rho correlation analysis at a p-level of 0.05. Correlation was ascertained between grades for tests of competence and the corresponding clinical requirements. For example, clinical requirements completed in operative dentistry were compared with SCOT I scores. A value of zero was recorded in the dataset for students who did not complete any category of clinical requirements. These were not considered missing values and were not removed or substituted with a proxy value for the analysis.

## Results

The mean numbers of clinical requirements and grades attained in tests of competence are presented in Tables 1 and 2 respectively. Composite restorations were the most common procedures performed by dental students, ranging from 25 to 78 completed restorations. This number represented both simple and complex restorations. This was followed by scaling and root planning procedures, by quadrant, and anterior root canal treatment as the most common procedures completed by dental students. A review of the retrospective data revealed that fixed prosthodontics and posterior endodontics were the least common procedures completed by dental students. There were 18 students with no experience completing posterior endodontic procedures, 1 student with no clinical experience completing anterior endodontic procedures and 21 students with no experience completing procedures for fixed prosthodontics. The Kolmogorov-Smirnov test of normalcy revealed that the variables for the number of anterior and posterior endodontic procedures and the number of fixed prosthodontic units completed were not normally distributed and the null hypothesis that data was normally distributed had to be rejected, thus a non-parametric Spearman’s Rho test for correlation was used for further analysis.

**Table 1:** Mean (SD) Number of Clinical Requirements

Clinical Requirements	Mean (SD)	Range
Composites	47.7 (13.26)	25–78
Anterior Endodontics	5.01 (2.62)	0–12
Posterior endodontics	1.42 (1.08)	0–4
Scaling and Root Planning (per quadrant)	16.72 (7.49)	5–53
Units of Crown and Bridge	2.02 (2.02)	0–9

**Table 2:** Mean (SD) Grade for Tests of Competence (Represented as a percentage)

Test of Competence	Mean (SD) Grade (%)
SCOT 1	62.75 (10.58)
SCOT II	59.88 (8.88)
SCOT III	61.11 (8.66)
CAP	54.04 (5.71)

Table 3 shows correlation coefficients between clinical requirements and the corresponding test of competence together with statistical significance. There was a moderate degree of positive correlation between the number of anterior endodontic procedures and fixed prosthodontic procedures and associated tests of competence while there was a weak correlation for operative procedures, posterior endodontic procedures, periodontic procedures and the associated tests of competence.

**Table 3:** Spearman’s coefficients with two-tailed significances between clinical requirements and grades in tests of competence. (p-values denoted with a superscript * were statistically significant at a p-value = 0.05)

	Spearman’s Coefficient	p-value
No of composites* SCOT I grade	0.31 (weak correlation)	0.006*
No of posterior endo* SCOT II grade	0.31 (weak correlation)	0.005*
No of anterior endo* SCOT II grade	0.42 (moderate correlation)	< 0.001*
No of Crown and bridge units* SCOT III Grade	0.51 (moderate correlation)	0.654
No of Scalings and Root Planings * CAP Grade	0.23 (weak correlation)	0.040*

## Discussion

Miller’s assessment pyramid describes the assessment of the performance of learners in unstandardized settings as the highest form of assessment [8]. Clinical settings where there is interaction with patients may be considered unstandardized workplace settings where competency assessments can take place and are most relevant [9]. However, dental educational institutions have struggled to implement competency-based assessments despite adopting competency-based educational frameworks. The competency-based examinations (SCOTs and CAP) described in the preceding section aim for dental students to perform a dental-related professional task at a suitable level of competence with minimal or no guidance from faculty assessors.

Within competency-based frameworks, assessment for learning should be the foundation of the assessment strategy over specified periods. This ensures that learners adopt a self-assessment approach to identify gaps in knowledge, skills, and attitudes and institute self-corrective measures for filling these deficiencies. Completing minimum clinical experiences provides opportunities for learning through in-action and on-action reflection, self-assessment and incorporation of faculty feedback into subsequent patient encounters. This ongoing and continuous process of complex learning, as students aim to achieve global competencies in the management of patients, may be the reason for the statistically significant positive correlation between the completion of clinical requirements and grades achieved in tests of competency.

This explanation, however, does not completely clarify the attainment of global competence since a statistically significant weak correlation was found between the number of posterior endodontics procedures completed and performance in tests of endodontic competence, despite the relatively low mean numbers of procedures completed. Additionally, the mean clinical requirement for fixed prosthodontics was comparable to the absolute minimum clinical requirement of two completed units, however, there was a moderate correlation that was not statistically significant with the corresponding test of competency. Even with operative procedures where the mean number of procedures performed far exceeded the minimum clinical requirements, only a weak correlation was noted. These mixed results are comparable to work carried out by Chambers who concluded that performance in independent tests of clinical skills was unrelated to the number of procedures previously completed [10].

In medical literature, the concept of competence and competency are often erroneously used interchangeably [11]. In dental education, competence may be considered an array of capabilities across affective, cognitive and psychomotor domains and related to task execution in a specific context. Conversely, competency can be considered as an individual’s transition towards expertise [12]. The framework of competency-based education adopted at this school in the English-speaking Caribbean measures or assesses competence in routine tasks expected of a beginner dental professional in these various tests of competence (SCOT I, II, III and CAP) while at the same time providing an opportunity for the transition towards expertise by the holistic management of comprehensive patients where minimum clinical requirements are met.

What is apparent from this analysis of retrospective data is that 22% of students had no experience in posterior endodontic procedures while 25% of students had no experience in fixed prosthodontics before tests of competence were taken. This dental school allows students to complete minimum clinical requirements after the final examination but before graduation. This potentially can put students at an educational disadvantage before such gateway assessments, particularly since positive relationships have been noted between clinical activity in fixed prosthodontic procedures and clinical dental student confidence [13]. This finding aligns with the work of a recent scoping review of dental schools that follow competency-based curricula in the United Kingdom where shortages of suitable patients to meet the demand for clinical experiences in complex restorative procedures have been reported [4]. This shortfall in clinical experiences is common in other schools of dentistry, where the most identified skills deficiency are in molar endodontics and fixed prosthodontics [14]. Ensuring minimal clinical requirements ensures the development of a safe dental practitioner in the context of future patient management. Further research is required to ascertain why minimal clinical requirements are not being met. Qualitative research is also planned to ascertain the factors that students believe are important in achieving competence in a range of clinical procedures.

## Conclusion

The null hypothesis was rejected since a positive correlation was found between the absolute clinical requirements completed and grades in tests of competence. However, only a weak to moderate degree of correlation was found between the completion of clinical requirements and performance in tests of competence for common clinical procedures that new dental graduates should be able to perform.

## Data Availability

The data that support this work can be found at Harvard Dataverse. 10.7910/DVN/QONYHR.
